# The Effect of Selected Factors on the Strength of Stitches of Upholstery Faux Leather

**DOI:** 10.3390/ma15196585

**Published:** 2022-09-22

**Authors:** Anna Vilhanová, Nadežda Langová, Robert Kłos, Eliška Máchová

**Affiliations:** 1 Department of Furniture and Wood Products, Faculty of Wood Sciences and Technology, Technical University in Zvolen, T. G. Masaryka 24, 96001 Zvolen, Slovakia; 2 Department of Furniture Design, Faculty of Forestry and Wood Technology, Poznan University of Life Sciences, Wojska Polskiego 38/42, 60-627 Poznan, Poland; 3 Department of Furniture, Design and Living, Faculty of Forestry and Wood Technology, Mendel University in Brno, Zemědělská 1665/1, 613 00 Brno, Czech Republic

**Keywords:** seam efficiency, strength of stitches, upholstery covering materials, faux leather

## Abstract

With their properties, upholstery covering materials significantly influence the quality of upholstered products. These materials form the surface layer of upholstered furniture; any damage to this material is immediately visible to the user. We consider the stitches connecting the covering fabrics to be one of the critical points of the upholstered surface, therefore they must have the required strength. Faux leather is one of the most used upholstery materials. The main aim of the paper is to determine the effect of upholstery faux leather, stitch length, point needle, and needle size on the strength of the stitches and the seam efficiency. The results of the experiment proved the suitability of using a sewing needle LR for joining covering materials such as faux leathers. The highest force to seam a rupture in the joints was achieved in the direction of the warp yarns of the underlying layer of PU (Polyurethane) faux leather with a stitch length of 4 mm and needle type LR/90. The highest seam efficiency was achieved with seams in the direction of the weft yarns of the PVC (Polyvinyl chloride) faux leather underlying layer with a stitch length of 4 mm and needle type LR/90. If the underlying layer of faux leather is a fabric with canvas binding, a higher seam efficiency of joints is assumed.

## 1. Introduction

The upholstery fabric leaves a first impression on the user, so it is essential that it meets broad quality requirements. When selecting the upholstery fabric, the way of upholstering, the nature of the pile, the Martindale rating, and the fire resistance must be taken into account. Factors that affect the comfort and quality of using the covering textiles include not only hygienic and aesthetic properties but especially their mechanical properties. In addition to the comfort characteristics of upholstery, the strength of the covering material, resistance to abrasion, and pilling are the most important quality factors of upholstered furniture. Covering materials used for furniture upholstery are sheet materials that need to be divided according to the required cutting plan and then joined again with stitches. The construction and material composition of the upholstery material affects not only the quality of the upholstered surface, but also the mechanical and appearance properties of the stitches. Composites from woven and knitted fabrics made of polyester multifilament fibres are most used due to the relatively low cost and good properties. Such composites also include various types of artificial leather. For these materials used in upholstery, we do not have sufficient information on the influence of basic factors on seam strength. The supporting layer of this material is made of polyester yarns. The most important features of polyester fibres are good durability and dimensional stability, high strength, easy maintenance, fast drying, low moisture adsorption, resistance to most chemicals and resistance to high temperatures. Rapid technological development, interdisciplinary research and cooperation between the science and industry have enabled the development of new materials in composites for furniture upholstery. The covering materials are almost exclusively joined by stitches–seams. The location and size of the load of the furniture construction must be considered when the stitches are created and positioned on the cover of the upholstered product. The seam strength of the covering material is often lower compared to the seamless areas of the cover. The quality and textile product serviceability loaded with operational forces usually depends on the strength and quality of the stitches, as well as on the properties of the fabrics [1,2].

To ensure product quality, manufacturers of upholstered furniture are interested in assessing the quality of joints of upholstery material. There is only a small amount of scientific work that comprehensively deals with the influence of several parameters on the strength of sewn joints of upholstery fabrics. Information regarding the quality of the stitches is more from the field of fashion textiles. The works [3,4] are focused on seam creasing, evaluation, and prediction of its quality. Relationships between yarn tension, types of sewing threads and seam quality, the impact of fabric parameters and properties on a 2D cutting and stitching line are presented in work [5,6]. Scientific works investigate the effects of the type of stitches on the strength of the sewing, the effect of the mechanical properties of cotton fabrics on the quality of the sewn joints, while claiming that the quality of the sewn joints depends on the strength, stretchability and density of the fabric being joined [7,8]. To minimize the deformations in the fabric in the seams and to keep the seam strong and durable if possible, it is necessary to select an appropriate sewing thread, stitch length, and other parameters such as needle characteristics, thread tension, and stitch type [9,10,11]. The study [12] is aimed at the static tensile tests for selected upholstery fabrics and the identification of the most optimal connections for fabric and stitch. The conclusion was drawn that the fabric tensile strength was the highest for Secret 10 fabric. In addition, the strength of upholstery covers is not influenced by the direction of the fabric die cut. The used method implemented for upholstered furniture allows for an objective assessment of the strength of upholstery covers and the selection of the most advantageous fabric–seam combination for future furniture designs. The choice of the type of stitches and the factors that affect their quality can be expressed using the seam efficiency of the joint. Compared to cotton threads, polyester threads achieve higher seam efficiency. In addition to the strength properties of stitches of fashion textiles, it is also important to recognize their elastic and deformation characteristics, and it is necessary to seek and create suitable methods for their evaluation [13]. When attaching fabric to complicated shapes and structures, different stitching techniques are employed. The researchers have reported that the core spun thread gives higher seam efficiency as compared to the conventional sewing thread and that the use of silicon finish decreases the seam efficiency. The tensile strength of the woven fabric not only depends on the strength of the constituent yarns, but also on the fabric geometry, warp and weft density, weave designs, yarn interlacement pattern, weaving conditions as well as fabric finishing treatments. Geršak reveals that tensile strength of woven fabric is decreased by the application of the sewing operations, and the ultimate serviceability of a sewn article depends on the technical features of the fabric as well as the seam [14,15]. The authors [16] pointed out that the fireproofing of fabrics changes the seam strength, which may be due to fabric stiffening, resulting in less fabric flexibility. In their research they aimed to determine the influence of thread type on the tearing strength of seams. The scientific work [17] studied a challenging example of an uneven appearance of the two-needle decorative seams of leather seat covers. Four natural and two artificial leathers were tested in the study. It was confirmed that the uneven appearance was influenced by the type of leather, the point of the sewing needle and the sewing thread.

One of the main sewing parameters, directly related to artificial leather sewability and quality of final product, is the needle penetration force (NPF). This force affects the initial deformation of the sewn joint and the size of the hole in the upholstery fabric. The results showed that the effect of backing fabric structure on NPF is significant. The highest NPF values were observed in woven fabric reinforced samples, followed by those reinforced with knitted and then nonwoven fabrics. It was observed that effect of needle point type on NPF is not significant. Moreover, it was seen that finer needles enhanced sewability in terms of reducing the amount of force exerted on the artificial leather [18]. Needle penetration force also affects needle heating in polyester blend upholstery fabrics, higher sewing speeds can cause burnt spots on the fabric, lower seam strength, and a decrease in production due to thread breakage [19,20,21].

The finite element method was used for stitch analysis [22]. The analysis of the thread tension demonstrates that the upper and bottom thread tension mainly influences the formation of the current stitch, and the force cannot cause redistribution or slippage of the previous, already build stitches. The FEM model was tested at different friction coefficients and foam stiffnesses and the simulations demonstrate logical results, comparable to the analytically predicted one. The numerical characterization of the mechanical behaviour of a vertical spacer yarn in thick warp knitted spacer fabrics was processed in the study [23]. In addition, some scientific studies also deal with the analysis and effect of the inner layers of the upholstery system on the quality of the covering layers of the upholstered furniture [24,25,26,27]. One of the important properties for evaluating the quality and safety of upholstered furniture is flammability, which is described in scientific papers [28,29,30,31].

The function of the seam is to provide a uniform transmission of loads between two joined materials and to keep their integrity, which is not completely possible with a stitched seam [32,33]. When using upholstered furniture, the joints on the functional surfaces of the covering materials are subjected to tension. Tensile strength perpendicular to the seam is the basic mechanical property of stitches of covering material. The main aim of the paper is to determine the effect of upholstery faux leather, stitch length, point needle, and needle size on the strength of the stitches and the seam efficiency and establish recommendations for manufacturers of upholstered furniture that use faux leathers. The experimental techniques and approach for the mentioned tests were designed based on the standard procedure and testing equipment.

## 2. Materials and Methods

### 2.1. Material of Upholstery Covers

**Figure 1:**
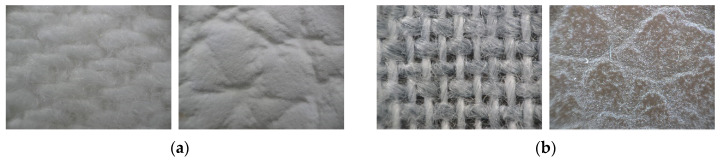
Types of tested faux leathers (150× magnification); left back side, right top side (**a**) PU faux leather fabric, (**b**) PVC faux leather fabric.

Material composition (textile construction) can be characterized as a two-layer material. The strength of the stitches was analysed on the two types of upholstery faux leather with different densities and different base layers. The reverse side of the covering material is the underlying textile with the mechanical properties, and the front side is made up of a layer of synthetic polymer. Material A is PU (Polyurethane) faux leather fabric (Figure 1a) with a base layer of pressed fabric from 100% PES (Polyester), and the surface, facing layer is made of PU. Material B is PVC (Polyvinyl chloride) faux leather fabric (Figure 1b) with the base material 100% PES with a folded twill weave and the surface layer is a PVC coating.

The highly strong and durable 100% polyester sewing thread Synton 30 was used as a connecting material. This thread is most often used when sewing upholstered furniture covers. The strength of the sewing thread is 28 N; its longitudinal elongation is 12% at rapture.

### 2.2. Type of Sewing Needles

**Figure 2:**
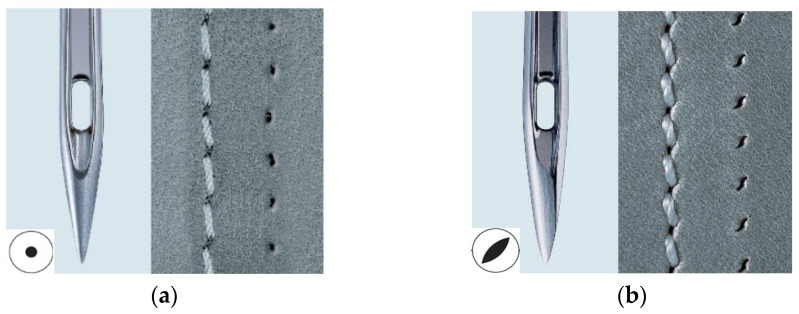
(**a**) tip of the needle R, (**b**) tip of the needle LR.

The needle point is the “point” or tip of the needle. There are different needle points available, which will determine both the appearance of the hole in the material and the appearance of the stitch. Two types of Groz-Beckert sewing needles, needle size 90/14 and 100/14, were used to create stitches. The metric sizing system corresponds to the diameter of the needle in the hundredths of a millimetre. Size 100 is one millimetre thick. The R-Style needle was the type used (Figure 2a). R point needles are known as universal or round point needles, used for any general-purpose sewing. R point needles are the standard for lockstitch machines and are commonly used for woven fabrics and soft leather. R point needles will produce a slightly angled or irregular stitch. The thread will be slightly elevated. A sewing needle with an LR tip was the second type used (Figure 2b). LR point needles cut the leather to the right at a 45-degree angle in the direction of the sewing, producing a slight to medium slanted stitch formation. The sewn thread is slightly elevated and stitch holes are visible. LR point needles are commonly used for decorative seams on footwear, leather bags, leather garments, and anything from soft to medium/hard leather.

**Figure 3:**
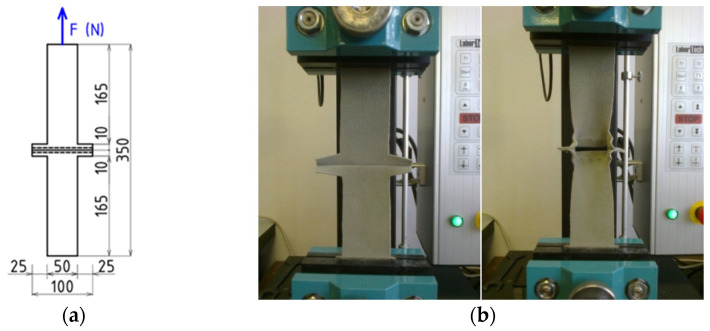
(**a**) the sample dimensions, (**b**) testing of the stitch.

The shape and dimensions of the samples (Figure 3a) were made according to ISO 13935-1:2014 [34]. The samples were created so that we could determine the effect of fabric type, needle size, point needles, and seam length on the strength of the stitch strength. For each type of covering material, stitches in both the warp direction and the weft direction of the base layer were created. We used two needle sizes, 90 and 100, to create a stitch. The point needles R and LR for each needle size were used. The stitches were made with a two-thread tying stitch, with a stitch length of 4 mm and 5 mm. We applied only these two lengths of stitches, because these are recommended for stitching leathers covering material, and yields the best stitch quality as well. Stitches with a length of 3 mm are too small and cause early tearing of the material under load. The longer the stitch, for example a stitch length of 6 mm, the looser the seam. Stitches with long stitch lengths hold the fabric together with less tension. The stitches were made on a Juki industrial sewing machine, with a tying stitch at a sewing speed of 4000 stitches/min. 

To determine the maximum force to rupture the seam, the Strip method was used, according to ISO 13935-1: 2014. The tests were performed using the universal numerically controlled testing machine LabTEST 4.05 (Test&Motion, Labor Tech, Praha, Czech Republic). During the tensile test, the clamping length was set to 200 mm ± 1 mm, and the loading speed was 100 mm/min. The maximum force was recorded, and the type of damage of the sample was evaluated during the test. The samples were prepared in both the basic and the weft direction of the covering materials. The joints were loaded with a tensile force perpendicular to the seam line. The maximum force to rupture the seam was determined. The loading scheme of the sample and the performance of the test are shown in Figure 3b.

The strength of the fabric must be determined to evaluate the effectiveness of the stitches. The dimensions of the reference samples for determining the strength of the fabric are the same as the samples with stitches. Additionally, the methodology of loading and determining the maximum force and textile rapture was the same. The efficiency of the stitch *P_S_* is determined following the Equation (1):

(1) $$P_{S}=\frac{F_{\mathrm{Smax}}}{F_{\mathrm{FLmax}}}\cdot 100 [\%],$$

where: *F*_Smax_ seamed fabric strength (N), *F*_FLmax_ unseamed fabric strength (N).

## 3. Results

The maximum force to seam rupture force *F*_Smax_ (N) for seamed samples were measured to evaluate the tensile properties of the seams. The average rupture force of reference samples of unseamed faux leather *F*_FLmax_ (N) are shown in Table 1.

Average values of maximum force to rupture the seam *F*_Smax_ using the strip method are shown in Table 2. 

Following Duncan’s test (Table 3 and Table 4), the effect of selected factors on the strength of the stitches was determined. In the case of PU faux leather, an effect of 100% of all monitored factors on joint strength is shown. Moreover, the effect of all three factors acting simultaneously is statistically significant at the 5% significance level (*p* = 0.00475). In the case of PVC faux leather, the effect of all three factors on the bond strength of the joint is 100% (*p* = 0.000). 

## 4. Discussion

### 4.1. Evaluation of Strength

The reference samples of unstitched faux leather achieved the highest strength in the direction of the warp yarn of the underlying layer. The average strength value of PU faux leather in the warp direction is 37.5% (1008.14 N) higher than that of PVC faux leather (630.08 N). In the weft direction of the base layer, the average strength value of PU faux leather was higher by 35.34% (699.32 N) compared to PVC faux leather (452.24 N).

The highest average values of the force to seam rupture were achieved by PU faux leather, with a stitch length of 4 mm and a needle type LR/90. Stitches generally achieved higher seam rupture forces in the warp direction; the highest breaking force was achieved with PU faux leather. In the warp direction, with a stitch length of 4 mm and a needle type LR 90, the breaking force was higher by 17.40% (521.92 N) than that of PVC faux leather (431.07 N). In the weft direction, with a stitch length of 4 mm and a needle type LR 90, the breaking force was lower by 1.16% (426.36 N) than that of PVC faux leather (431.06 N), which is a statistically non-significant difference. Moreover, the study [12] confirmed that, in general, the strength of the seam is higher in the warp direction.

Seam efficiency is defined as the capacity of the material to carry a seam. In general, the efficiency of the seams is 60–80%. Low seam efficiency values indicate that the stitched material will already be damaged during sewing. In general, the tested covering materials and the stitches achieved higher strength to seam rupture in the warp direction of the underlying layer of faux leather. The finding can be explained as the warp yarns of fabrics almost always have a higher strength than the weft yarns. This can be achieved, e.g., using the long and thick fibres in the construction of the yarn and by a more even structure of the fabric in the direction of the warp. 

The seam efficiency values (Table 5) of the tested joints of artificial leather fabrics ranged from 34.04% (PU faux leather fabric, in the warp direction, stitch length 5 mm, R/90) to 95.32% (PVC faux leather fabric, in the weft direction, stitch length 4 mm, LR/90). The highest seam efficiency was achieved in the direction of the weft yarns of the underlying layer with both faux leather covering materials, with type needles LR/90 and a stitch length of 4 mm. Seam efficiency of the PVC faux leather (95.32%) was the highest, 33% more than PU faux leather (60.96%).

### 4.2. Evaluation of Deformations and Damage

Since the warp yarns are stronger in both types of faux leather, the deformation in the direction of the warp fibres was also smaller. This was evident in the case of stitches, where the lowest deformations occurred in the case of PU faux leather fabric. With an LR/90 needle and a stitch length of 4 mm, the deformation was 26.47 mm, with a stitch length of 5 mm, the deformation was 22.76 mm. On average, 33% larger deformations occurred in the case of PVC faux leather, where with an R/100 needle and a stitch length of 4 mm, the deformation was 33.47 mm, and with a stitch length of 5 mm, the deformation was 32.19 mm.

Deformations are higher when stressing in the weft direction, this is due to the flexibility of the weft yarns, as well as their lower strength. The lowest deformations were achieved with PU faux leather fabric joints. With a needle type R/90 and a stitch length of 4 mm, the deformation was 47.31 mm, with a stitch length of 5 mm, the deformation was 45.99 mm. On average, 16% higher deformations occurred with PVC faux leather, where with an R/90 needle, and a stitch length of 4 mm the deformation was 57.27 mm, with a stitch length of 5 mm the deformation was 50.68 mm. The measured values showed that longer stitches of 5 mm are more suitable. It is a seam with a lower density of stitches, which causes less damage to the underlying layer of the covering material.

**Figure 4:**
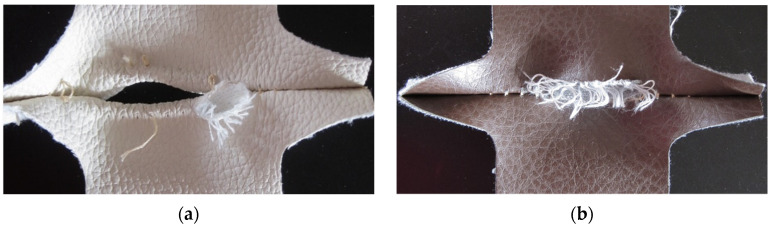
Seam slippage (**a**) PU faux leather fabric; (**b**) PVC faux leather fabric.

When stitches are stressed in the direction of the warp fibres, seam slippage is visible. The yarn in the underlying fabric was pulled out of the seam along the edge. With this type of deformation, the weft yarns in the underlying fabric are separated, the weaving of which has a higher elasticity. This type of damage was more pronounced in the stitches of the PU faux leather fabric covering material. When stressing the stitches of PVC faux leather fabric, the warp yarns of the underlying fabric ruptured when the maximum force was reached. The characteristic seam slippage damage is shown in Figure 4.

**Figure 5:**
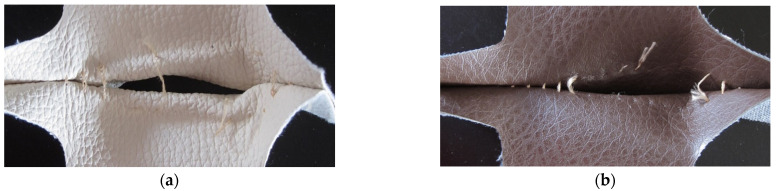
Seam grinning: (**a**) PU faux leather fabric; (**b**) PVC faux leather fabric.

Seam grinning occurred when stitches were stressed in the weft direction. A gap was created in the stitches, exposing the sewing thread, which tore when the maximum force was reached. The damage was also manifested by the enlargement of the hole into which the needle penetrated. It can be also seen in the case of a stitch length of 4 mm, i.e., with reduced seam density, seam grinning increases. Sewing thread in a seam that is loaded perpendicular to the stitch line tends to tear. The characteristic damage of seam grinning is shown in Figure 5. The results of our research are comparable with the study [12], where the seam failure is determined by the direction of the specimen’s die-cut. In both fabric types, the weft and warp are significantly different; hence, the variable form of destruction is closely related to the cutting direction of the samples.

## 5. Conclusions

Stitches and seam types are very important when evaluating the upholstery quality. Stitches are used to join the patterns of the upholstery, and seams give the shape and detail of the upholstery. The types of fabrics and their structural properties have a significant impact on seam efficiency. When using faux leather as covering upholstery materials, it is necessary to consider the type of the underlying layer of the textiles in terms of the seam efficiency and its damage and defects. If the underlying layer of faux leather is a fabric with canvas binding, a higher seam efficiency of joints is assumed. 

In terms of the strength of the stitches and the influence of all investigated factors, the joint with a stitch length of 4 mm is the most suitable for PU faux leather in both the warp and weft directions of the underlying layer. For PVC faux leather in the direction of the warp and weft yarns of the underlying layer, a stitch with a length of 4 and 5 mm is suitable. The difference in the strength of the seams is minimal, which is caused by the uniform structure of the underlying layer in both directions. 

In terms of seam efficiency, it is recommended to use the LR needle for sewing faux leather. The seams achieved higher efficiency in the case of both types of covering materials. 

When testing both types of covering materials, the highest seam efficiency of 95.32% was achieved for PVC faux leather joint. The higher efficiency of PVC stitches indicates the appropriate selection of all factors affecting the stitches. Since the factors affecting the seam efficiency are the same for both covering materials, whereby the efficiency of PU faux leather stitched joints is lower, we recommend experimentally verifying the effect of another factor, the fineness of the sewing thread. 

If it is possible in terms of the cutting plan and the construction of the covering fabric, the stitches should be made in the direction of the warp yarns. In this direction, the strength of both the fabric and the joint is higher.

## Figures and Tables

**Table 1:** Average values of unseamed fabric strength *F*_FLmax_ (N).

Material A: PU Faux Leather Fabric		Material B: PVC Faux Leather Fabric	
Load in the warp *F*_FLmax_ (N)	Load in the weft *F*_FLmax_ (N)	Load in the warp *F*_FLmax_ (N)	Load in the weft *F_FLmax_* (N)
1008.14	699.32	630.08	452.24

**Table 2:** Average values of seamed fabric strength *F*_Smax_ (N). * The standard deviation (SD) is given in brackets.

Point Needle/Needle Size	Material A: PU Faux Leather Fabric				Material B: PVC Faux Leather Fabric			
	Load in the Warp Direction		Load in the Weft Direction		Load in the Warp Direction		Load in the Weft Direction	
	Steam Length (mm)		Steam Length (mm)		Steam Length (mm)		Steam Length (mm)	
	4 mm	5 mm	4 mm	5 mm	4 mm	5 mm	4 mm	5 mm
R/90	463.82 (SD = 9.05) *	343.21 (24.02)	386.46 (20.76)	400.44 (10.38)	362.46 (16.55)	407.75 (11.01)	408.11 (11.68)	347.00 (18.01)
R/100	492.97 (24.56)	456.12 (20.73)	380.60 (13.46)	391.32 (16.32)	357.01 (16.54)	400.00 (5.72)	400.05 (15.80)	339.12 (5.52)
LR/90	521.92 (14.25)	413.26 (14.76)	426.32 (22.46)	407.99 (14.83)	431.07 (8.17)	407.67 (18.64)	431.06 (18.13)	420.93 (17.66)
LR/100	517.12 (18.21)	409.19 (19.04)	424.52 (23.05)	407.43 (21.32)	412.11 (8.91)	410.67 (5.83)	411.4 25.96)	382.59 (7.70)

**Table 3:** Basic table of three-factor analysis of variance for the strength of joints of PU faux leather.

Source of Variation	Sum of Squares	DoF	MS	F	*P*
Intercept	20,494,153	1	20,494,153	44,469.82	0.000000
Load Direction	69,059	1	69,059	149.85	0.000000
Needle size	33,852	3	11,284	24.49	0.000000
Stitch length	66,029	1	66,029	143.27	0.000000
Load Direction × Needle size	21,630	3	7210	15.64	0.000000
Load Direction × Stitch length	58,473	1	58,473	126.88	0.000000
Needle size × Stitch length	11,788	3	3929	8.53	0.000045
Load Direction × Needle size × Stitch length	6352	3	2117	4.59	0.004755

**Table 4:** Basic table of three-factor analysis of variance for strength of joints PVC faux leather.

Source of Variation	Sum of Squares	DoF	MS	F	*P*
Intercept	17,651,343	1	17,651,343	56,459.80	0.000000
Load Direction	2044	1	2044	6.54	0.012129
Needle size	49,506	3	16502	52.78	0.000000
Stitch length	6196	1	6196	19.82	0.000023
Load Direction × Needle size	363	3	121	0.39	0.762619
Load Direction × Stitch length	17,933	1	17,933	57.36	0.000000
Needle size × Stitch length	1783	3	594	1.90	0.134563
Load Direction × Needle size × Stitch length	24,283	3	8094	25.89	0.000000

**Table 5:** Seam efficiency for Faux Leather Fabric.

Point Needle/Needle Size	Material A: PU Faux Leather Fabric				Material B: PVC Faux Leather Fabric			
	Load in the Warp Direction		Load in the Weft Direction		Load in the Warp Direction		Load in the Weft Direction	
	Steam Length (mm)		Steam Length (mm)		Steam Length (mm)		Steam Length (mm)	
	4 mm	5 mm	4 mm	5 mm	4 mm	5 mm	4 mm	5 mm
R/90	46.01%	34.04%	55.26%	55.26%	57.53%	64.71%	90.24%	76.73%
R/100	48.90%	45.24%	54.42%	55.96%	56.66%	63.48%	88.46%	74.99%
LR/90	51.77%	40.99%	60.96%	58.34%	68.42%	68.41%	95.32%	93.08%
LR/100	51.29%	40.59%	60.70%	58.26%	65.41%	65.18%	90.97%	84.60%

## Data Availability

The data presented in this study are available on request from the corresponding author.
